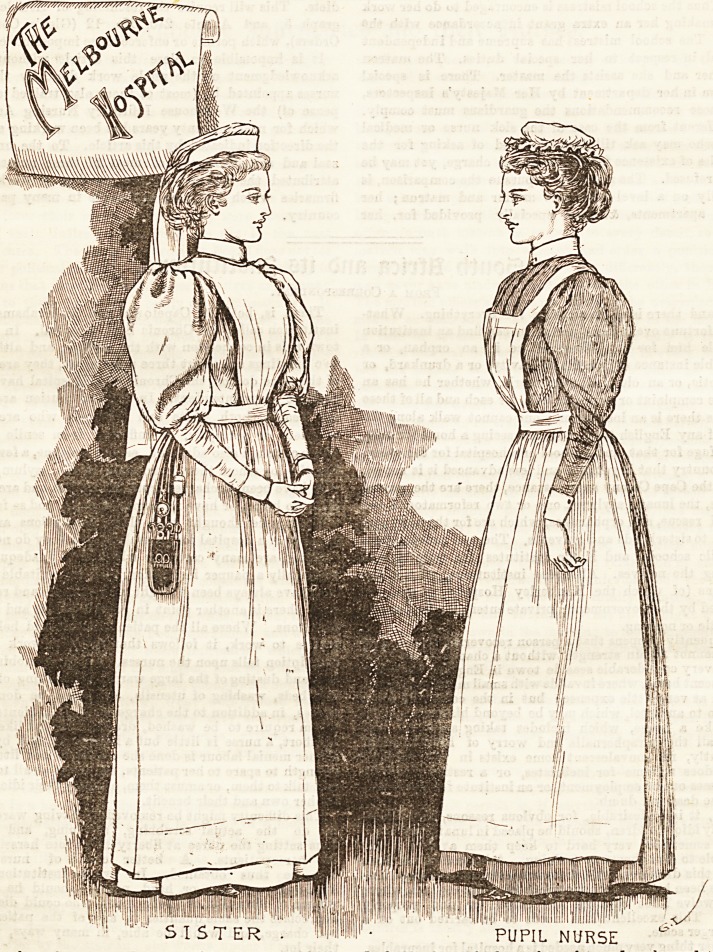# "The Hospital" Nursing Mirror

**Published:** 1896-09-19

**Authors:** 


### "The Hospital" Nursing Mirror.

Being the Extra Nursing Supplement of "The Hospital" Newspaper.

[Contributions for this Supplement should be addressed to the Editor, The Hospital, 428, Strand, London, W.C., and should have the word "Nursing" plainly written in left-hand top corner of the envelope.]

### News from the Nursing World.

### H.R.H. PRINCESS CHRISTIAN.

H.R.H. Princess Christian, as President of the Royal British Nurses' Association, is evincing much activity in its interests at the present time. Princess Christian will present badges to members at the annual conversazione in December, and has further expressed her intention of attending some of the practical demonstrations on invalid and convalescent cookery, to be given at 17, Old Cavendish Street, W., during the coming winter.

### HOSPITAL STORIES AT THE PEOPLE'S PALACE.

The East London Exhition at the People's Palace, Mile End Road, has been such a success that it is to be kept open till the beginning of November. Miss Gethen has been giving recitals of some of her original "Hospital Stories," which have proved so popular that she has been asked to continue them, and has consented to do so every Thursday throughout September and October at half-past five and seven p.m. The large Queen's Hall has been well filled on these occasions by audiences who have evidently much enjoyed the graphic little sketches of hospital life so well rendered by their author. Perhaps there are few dwellers round the People's Palace who do not know the interior of the great East End Hospital and a certain aspect of hospital life, but Miss Gethen's stories touch a chord less familiar to the world in general and show its bright as well as its pathetic side. Admission to the exhibition for the modest sum of threepence admits also to these recitations as well as excellent concerts and organ recitals.

### ST. SAVIOUR'S UNION INFIRMARY, DULWICH.

At a recent meeting of the St. Saviour's Board of Guardians, the following letter was read from Nurse Alice Marie Meadows, who has lately left the infirmary: "I wish to take this opportunity before leaving the infirmary to thank the guardians, medical superintendent, doctors, and matron for all that is done for the comfort and welfare of the nurses. The three and a-half years I have spent at the infirmary have been profitable and happy ones. Great pains are taken by the medical superintendent, doctors, matron, and the assistant matron in preparing the nurses for examination, and the training will bear comparison with the training schools of our London hospitals."

### NURSES WANTED FOR SHEFFIELD.

District nursing was first started in Sheffield in 1888, and the number of nurses in the town has now increased to thirteen, but Miss Corvin, lady superintendent of St. George's Home, Sandon Place, in a recent letter to the *Sheffield Daily Telegraph* asks: "What are these among so many?" Generous contributions have been given by several business firms in Sheffield, and by Mr. Bernard Firth, but more money is wanted to enable a nurse to be started in the Attercliffe district. The "lending" and "giving cupboards" in connection with St. George's Home are emptying, and sadly need replenishing with left-off clothes, old linen, and other necessaries. Miss Corvin's appeal to the public of Sheffield ought to meet with a ready response.

### THE CHOICE OF A TRAINING SCHOOL.

The general aspect of some of the nurses one meets in the course of daily walks abroad raises a wonder in the mind as to the reasons which have induced these wearers of cloak and bonnet to adopt the care of the sick as a profession. Untidy hair, fly-away veils, a pinched-in appearance about the waist, and high-heeled, pointed-toed shoes are painfully out of place in women who follow the calling of a nurse. Can it be possible that many women begin their education in nursing in the spirit of a would-be probationer who was heard the other day to exclaim, looking despondently the while at the rules and regulations of various training schools spread out before her, "You see, what I really want to find is a hospital where I may keep my fringe. So many of these matrons object to fringes, and I simply *cannot* sacrifice mine!"

### MALE NURSES IN PENNSYLVANIA.

Male nurses are likely soon to be in demand in Pennsylvania. The State Board of Charities is intending to recommend the establishment of training schools for male nurses in several parts of the State, and the substitution of men for women nurses in the male wards of all the hospitals which are supported by Government grants. They will also urge upon the Legislature the desirability of passing an Act of Assembly making it compulsory throughout the State that male patients shall be tended by male nurses only. In doing this it is proposed to replace the present class of hospital orderlies by well-educated and intelligent young men.

### NURSING AT ST. MARYLEBONE INFIRMARY.

The Guardians of the Parish of St. Marylebone, owing to the increasing requirements of their large separate infirmary at Notting Hill, have made arrangements to take into the Nurses' Home there six extra probationers. This home, which was opened in July, 1884, by H.R.H. Princess Christian, is in two wings, with a large lecture or class room in the centre. One block contains rooms for the night nurses, the other is set apart for the probationers in their first year. Each nurse and probationer has a separate bedroom. Courses of lectures are given to the probationers by the medical superintendent, Mr. Lunn, and bi-weekly classes by the "Home Sister," also instruction in massage. Certificates are given at the end of three years' training to those who have passed satisfactorily through their practical and theoretical work. Each probationer receives during her first year £10, with indoor uniform, the second year the salary is raised to £20, and afterwards by yearly increments to £25. The salaries of a certain number of the probationers in their first year are paid by the committee of the Nightingale Fund, this committee also granting gratuities in the second and third years to those probationers whose work during each year has been considered satisfactory.

### NURSING IN INDIA.

For the benefit of those who are frequently asking for particulars as to nursing abroad, we quote from a letter that has recently reached us from a sister in the Indian Nursing Service, who says: "We have read letters which infer that nursing here is all work and no play, but those you have published during the last year have been true to life!" This is a useful and unsolicited criticism which will be helpful to nurses when contemplating service in India.

### TO HELP THE CHILDREN.

A sale of work in aid of St. Monica's Home Hospital for Children, Brondesbury, is to take place in November at the rooms of the National Health Society, under the patronage of the Duchess of Bedford. Contributions towards the sale will be very gladly received by Miss Forster, at the home; and on any afternoon except Sundays and Fridays visitors who may like to inspect the home will receive a cordial welcome from Miss Marshall, the lady superintendent.

### THE CLEANING SEASON.

Cleaning operations are in full swing throughout London, and house painters and whitewashers are having a good time. The hospitals are no exception to the rule, and repairing and cleaning is going on as busily within their walls as in private houses. Amongst others, at King's College Hospital, as at Middlesex, workmen are busy. The New Hospital for Women is given over into their hands, and the Mildmay Mission Hospital has been closed for some weeks on the same account.

### VACCINATION IN AFGHANISTAN.

Miss Lilias Hamilton, M.D., private physician to the Ameer of Afghanistan, is introducing calf-lymph vaccination into that country, where at certain times of the year small-pox rages. The principle of vaccination being explained to the Ameer, he forthwith decided to establish vaccine stations, and invited his subjects to consent to the universal vaccination of children, giving orders for two calf stables to be built according to the model taken back by Miss Hamilton, after her visit to England with the Shahzada last year. Miss Hamilton is the only European physician in Afghanistan, and her labours in the dispensary she has established in Cabul, where she is assisted by an English trained nurse, are greatly valued.

### NURSING AT SCULCOATES WORKHOUSE.

One of the nurses at this infirmary has been making certain statements with regard to the condition of the nursing, which were rightly considered by one of the guardians at a recent meeting of the Board to require explanation. Nurse Walsh complained that only probationers were left in charge of dying patients, three nurses being off duty at one time, and that the night nursing was altogether insufficient. The chairman of the House Committee, in reply to Mr. Lilley, stated that a thorough investigation had been made into these charges, but, according to local reports, the "explanation" offered, which appeared to satisfy the Board, seems very incomplete. "No nurses were off duty other than allowed by the rules of the Board, therefore there was no blame to be attached to the superintendent nurse;" but this does not affirm that there was no blame to be attached to those who were responsible for the insufficient number of the nurses. With regard to the other statement, "if there were not sufficient nurses for night duty it could not be done. There was at present only one nurse to put on night duty." That is to say, if there are more patients than the existing staff can attend to, they must be neglected. They are only paupers—what does it matter!

### THE AUCKLAND HOSPITAL.

The troublous times through which the Auckland Hospital has been passing of late are thus alluded to in the annual report of Dr. MacGregor, inspector of the hospitals in the colony: "The last has been a year of great turmoil at this institution, during which the evident want of discipline among the nurses culminated in a change of system. Dr. Baldwin has been appointed medical superintendent and Miss Squire matron. I have long known Miss Squire to be a capable, energetic woman, and Dr. Baldwin comes with a good record. Both have certainly undertaken a heavy task in restoring efficiency and discipline in this institution. Among the nurses a spirit of insubordination has been prevalent, and a constant tale-bearing to persons outside. . . . The matron ought to have an office in the hospital, and her authority should be upheld in every way." Under the new *régime* there will, we hope, succeed a time of peace and good government. The friends of the Auckland Hospital have lately been busily occupied in getting up a ball, the proceeds of which are to go towards furnishing the new wing now being added to the nurses' home.

### LADY DOCTORS IN TURKEY.

We hear that the Sultan of Turkey has prohibited lady doctors from attending upon his subjects, and that Dr. Grace Kimball, who had established herself with success in Turkey, and worked there for fourteen years, has now returned to London.

### KENT NURSING INSTITUTION.

The nurses of the Branch Home at Bromley are losing their Lady Superintendent, Mrs. O'Neill, and have presented her with a gold cable bracelet as a small token of their esteem and affection towards one whom they feel has shown them much kindness and sympathy.

### SHORT ITEMS.

It is said that nursing is becoming the fashion in France. Amongst other ladies who have lately taken up hospital work is Mdlle. Aynard, a daughter of the Deputy of Lyons. — The Stockton-on-Tees and Thornaby Nursing Association reports thirty-nine new cases during August, 1,030 visits having been paid in the course of the month.—The Nursing Association of Stanhope benefited to the amount of about £7 by the various collections on "Nursing Sunday."—A Nursing Association is being started in the village of Hessle, Hull. A general committee and a working men's committee have been formed, and it is proposed to make a house to house collection, in the course of which the smallest contributions will be gladly accepted.—Mdlle. Walborg Anderson, a leading singer in the Royal Opera House, Copenhagen, who is expected to visit London next year, was formerly a nurse in one of the hospitals at Copenhagen.

### Hygiene: For Nurses.

By JOHN GLAISTER, M.D., F.F.P.S.G., D.P.H.Camb., Professor of Forensic Medicine and Public Health, St. Mungo's College, Glasgow, &c.

### XXIV. — SANITARY FITTINGS (*continued*). — THE HOUSE-DRAIN.—TRAPS AND THEIR USES.

THE house-drain is that part of the soil-carrying apparatus which is situated between the intercepting trap at the bottom, or termination, of the soil-pipe and the common sewer in the roadway. Considerable care must be exercised in laying it, and it should be planned to have as straight a course as possible to the sewer. This is called the alignment. It must be laid with a downward gradient from the trap—its highest point—to the sewer—its lowest point—so that the drainage will flow from the result of gravity. When laid too level solids are liable to be deposited, "silt" is formed, and, sooner or later, the drain becomes choked. If, on the other hand, the slope downward be too great, the volume of descending water from the soil-pipe passing through the trap is apt to "siphon" the trap owing to the momentum—that is, the combined weight and velocity—of the passing column of liquid. The proper level is, therefore, adjusted by the eye of the experienced workman, or, better, by some instrument as figured in Fig. 37, which represents Moss-Flower's Gradient Indicator.

FIG. 37.—A, Brass-plate to indicate gradients; D C, Spirit level; D E, Drain-pipe; F G, Drain-limb of instrument for applying flush with pipe; H, Tangent screw, for very accurate adjustments.

The bed upon which the soil-pipe is laid ought to be composed of some immovable material to secure solidity and to prevent disjointing of pipes from uneven "settling" of house foundations, which is especially liable to happen in buildings founded upon "made-up" or "filled-in" sites. Concrete, or cement, forms the best bed. The house-drain is composed usually of either fire-clay or iron pipes, in lengths, each length being fitted into the other, and the joints made impervious by cement in the case of the former and oakum or tow and molten lead in the latter. The best constructed drain, however, is liable, upon occasion, to become blocked from negligence or carelessness either on the part of domestics or householders themselves, and the obstructing causes are of a very varied character; scrubbing-brushes, washing-cloths, dusters, and other kindred articles often cause obstruction which must be got rid of. Where the drain is closed in its entire length this can only be accomplished by passing a long flexible iron shaft along the pipe from a part which has been opened for the purpose. This, however, is an unsatisfactory and expensive method. Provision, therefore, in the original construction may be made for such a contingency by putting in, at intervals, sections of pipes, having movable lids, called "inspection pipes." Figs. 38, 39 represent these in position.

FIG. 38.—BUCHAN'S INSPECTION PIPE,

D C, Movable openings by which interior may be examined; C is placed at a junction with a branch, indicated by oval ring; L, Ordinary pipe length; M N P Q, Junctions of pipes.

A house-drain ought to possess the following points, viz. (1) It should be laid on a solid bed of concrete or cement: (2) it should have a sufficient gradient; thus, for a 4 in. pipe a fall of 1 in 40; (3) there should be no right-angled connections, but oblique, in the direction of the flow; (4) its interior should be as smooth as possible; (5) it should be automatically flushed; (6) it should be disconnected from the soil-pipe by an intercepting ventilating trap, which should not open on a pavement, or close to windows or doorways.

TRAPS.—Frequent use has already been made of the word "trap," and mention has been made of its being an essential part of the drainage system of the house. It is, therefore, necessary that explanation of its use be now given. The object of a trap is suggested in the word itself. It is an arrangement whereby something is arrested or trapped, which it is desirable to arrest or trap. In relation to sanitary fittings that something consists of foul air and gases generated in pipes, which carry foul and putrescible materials, and micro-organisms. The medium by which these are trapped is water more or less pure, the point of arrest being situated between the house interior and the nearest adjacent foul-pipe, or channel. If a glass tube be taken, and in a flame be bent twice in a round curve, and if, after it cools, the tube being held in a perpendicular position, water be poured into it, there will be formed a part of the tube in which water will constantly lie, and through which air at the ordinary pressure will not pass. We have thus formed the most elementary form of trap, consisting of a simple bend in a tube. Should air or gas attempt to pass from the lower end of the tube to the upper it is arrested or trapped by the column of water lying in the bend. Should the gas be passed under pressure, the trap would be forced; in other words, the force of the pressure would overcome the weight of the column of water in the bend of the trap. From this simple experiment and apparatus the essential features of a trap may be understood.

FIG. 39 shows Inspection-pipe with pedestal feet, laid on concrete bed.

If, however, a branch tube be blown in the glass model, at the top of the top of the upward bend, no ordinary pressure will force the trap, as the air or gas finds a vent by the ventilating-tube.

A more primitive form of trap, in use when circular drains were not thought of, is called the "dipstone" trap.

When, however, the circular or pipe form of drain came to be adopted, the trap had to partake of a character suitable to the new form. Hence the most elementary form, a simple bend, or a depressed tongue, in the pipe formed the trap. Fig. 40. illustrates the "dipstone" principle adapted to the

FIG. 40.—HONEYMAN'S SOMERSET TRAP.

A, Inlet opening of drain; B, Exit opening of drain; between them is the fixed column of water forming the trap, the depressed tongue acting as the "dipstone"; C D, Ventilating and cleansing "eyes" of trap.

circular pipe, which was patented by Mr. Honeyman, a Glasgow architect, in 1868.

The column of water which lies in the bend of the trap is called the water-seal or water-lock. Experience with traps in connection with pipes through which water flowed with considerable velocity, soon showed that the water-seal was liable to dislocation or "forcing" from different causes. The disturbing force was found to be either difference of atmospheric pressure or momentum of the flowing water; and the effect produced was the emptying, partial or total, of the water-seal. This action, from its parallelism to the action of the siphon came to be called the "siphoning" of a trap.

### Workhouse Infirmaries and their Nurses.

To go back a little into history. Trained nursing in hospitals may be said to have begun after the Crimean War, and if the year 1870 be put as the date of its general theoretical adoption, it will not be far out. Now as Government institutions go, this is quite a recent date; as guardians go it is also a new thing. Red tape and conservative notions prevail at the central office, conservative ideas and gross ignorance and incapacity prevail at the local centres presided over by the guardians. The Government Inspectors have no power to enforce their wishes, even if they were themselves trained for their duties—which they are not in the vast majority of cases. The Local Government Board does not see its way—for political reasons—to put its foot down and say to the guardians that they shall administer the lives and money committed to their charge in a really efficient manner. It merely sends out circulars to the boards, and these, for the most part, are never even read by those to whom they are addressed. The Local Government Board says to the guardians that they must perforce obey a set of "orders" which they have drawn up from time to time, but it makes the "orders" of such a character that the guardians cannot help fulfilling them if they want the workhouse infirmary to remain standing.

These wise "orders" enact that the master of the workhouse shall be the sole authority in the workhouse and infirmary, and that the matron shall have charge of the sick. The master need know nothing about sick people and the matron need only know how to keep accounts. The qualification for the nurse is that she should be able to read the directions upon the medicine. Such are the qualifications for those who have the responsibility connected with the nursing of every patient (they are technically called "not-able-bodied indoor paupers") inside the walls, which, in the greater number of cases, include both workhouse and workhouse infirmary. In the case of those infirmaries which are no longer under the control of the master, that is, which are separate buildings, the case is different, though still not satisfactory in many respects. Now, what well-trained nurse will undertake to nurse patients where they are primarily paupers under the repressive workhouse system, and, secondarily sick folk? The sickness is sunk in the pauperism. Ignorance and pauperism go together.

Let us see what are the actual conditions under which a nurse works in a hospital. She enters there, we will say, for her training, and after a year's time is placed in a more or less responsible position to the patients, but she is not considered to be fully trained till she has had three years' training, and in all manner of cases. She then passes a theoretical and practical examination and gets her certificate. While in the hospital she has her hours off duty, intercourse with educated persons, is well supervised by the "sister" and looked after by the matron; she works under the eye of the medical and surgical staff, and she has very few reports to make and very few vexatious rules to worry her. She has a certain position, a position which is becoming more and more highly thought of every year, and if she takes up all the branches of her work she can make a fair living for herself or a more secure, though, perhaps, less lucrative one if she joins one of the nursing associations. She has certainly to work very hard in hospital, and she has a great deal of strain consequent upon the necessity of keeping up to a good standard of conduct and of nursing.

Now compare her position with that which she will have if she elects to enter into the Poor Law service instead of into private or institution nursing. The moment she enters the infirmary she becomes a servant on a par with the porter and other subordinate officials. Her visitors are subject to the matron's approval; she has no special accommodation; her salary depends on the will of the board; she has no independence of action, and is subject to the untrained and often uncouth matron in respect to every detail, so that a matron may walk into a ward and order a poultice to be removed, or a dressing to be done differently, though she may never have made the one or done the other in her life. The medical officer's orders are liable to be overruled by the master or matron, for he has no authority in the infirmary. In case of severe or sudden illness, not only is there no resident medical officer, such as is found in every hospital, but the master is the person who has to send for medical assistance, and, of course, he can and does exercise his discretion and overrules the nurse if he thinks fit.

What educated or trained nurse will subject herself to such rules? What nurse will stand seeing the directions which she has received direct from the medical man in charge of the case overruled by an ignorant, untrained woman, a woman who usually belongs to an inferior class to herself?

The petty slights, the low surroundings, the insanitary conditions of life; the commonest and most necessary nursing requisites, *e.g.*, hot water and baths, too often noticeable only by their absence; the hours of relaxation of uncertain occurrence and short duration; the inferior food; the nursing of the patients by night too often totally wanting, and the nurse (if she has not voluntarily sat up all night with her case) may come down in the morning to find the result of her efforts of the previous day more than compensated for by the neglect during the night.

As to her labours, Hercules could not accomplish all she has to do, and the best assistants she can expect to get are the imbeciles—they make, it is found, the best helps in workhouse infirmaries—the only alternative being the incapable pauper helps.

Many of the nurses are untrained, and this puts additional work upon those who are trained, and the management of the chronic sick person is, if anything, more delicate than that of an acute case. The advanced cases found in workhouse infirmaries are never seen in general hospitals, and are trying in the extreme. The very monotony of the work in itself demands that the nurse should have a high standard to begin with, and good surroundings to keep it up. The reverse is the rule, and the approval and support the nurse requires is mostly absent.

When one turns to the acute cases which are often admitted into infirmaries—the typhoids, the pneumonias, the midwifery cases—one must admit that the infirmary nurse should be well trained. The well-trained nurse, however, soon gets sick of it all, and departs for ever from the Poor Law service.

From the economical point of view, the pauper assistant, and, next to him, the untrained nurse, is a very expensive luxury—the lives of paupers appear to be of very little economic value—and the rates are greatly increased by the waste these persons create. The extra expense of the trained nurse is soon made up for.

What is to be done to improve workhouse infirmaries? First of all encourage trained nurses to enter workhouse infirmaries to stay there. This implies that their position must be improved. It should be assimilated to that of the school mistress, to whom, by training and education, she is fully equal. Thus the school mistress is encouraged to do her work well by making her an extra grant in accordance with the results. The school mistress has supreme and independent command in respect to her special duties. The matron assists her and she assists the master. There is special inspection in her department by Her Majesty's inspectors, with whose recommendations the guardians must comply. How different from the case of the sick nurse or medical officer, who may ask till they are tired of asking for the necessaries of existence for their especial charge, yet may be as often refused. The mistress, to pursue the comparison, is practically on a level with the master and matron; her rations, apartments, &c., are specially provided for, her salary is independent of the will of the workhouse authorities. Her assistants must be proper ones. Yet the nurse who has charge of children when sick may be only able to get "improper" assistants.

No improvement will take place in these workhouse infirmaries till it be forbidden for any pauper to be employed as a nurse in the sick wards, and till the workhouse matron be relieved of her duty of taking charge of the sick and their diets. This will require the rescinding of Article 99, paragraph 5, and Article 210, No. 12 (Glen's Consolidated Orders), which permit or enforce these impossible conditions.

It is impossible to close this article without a brief acknowledgment of the noble work done by the trained nurses appointed by (most of them also trained at the expense of) the Workhouse Infirmary Nursing Association, which for nearly twenty years has been working steadily in the direction indicated by this article. To the unremitting zeal and self-effacement of most of these nurses must be attributed the advance in the nursing of workhouse infirmaries which has been noticeable in many parts of the country.

### South Africa and its Institutions.

FROM A CORRESPONDENT.

In England there is an institution for everything. Whatever misfortune overtakes a person he can find an institution to console him for it. Whether he is an orphan, or a remarkable instance of juvenile depravity, or a drunkard, or an epileptic, or an old man past work, whether he has an incurable complaint or a curable one, for each and all of these afflictions there is an institution; one cannot walk along the streets of any English town without seeing a home for this, and a refuge for that, and a school or a hospital for the other.

In a country that is younger and less advanced it is not so. To take the Cape Colony as an instance, there are the general hospitals, the lunatic asylums, one or two reformatories and homes of rescue, and orphanages, which are for the most part attached to sisterhoods and convents. Then, of course, there are public schools, and Kaffir institutes for training and educating the natives. All these institutions, with a few exceptions (of which the Kimberley Hospital is one) are supported by the Government; private enterprise has, so far, done little or nothing.

It frequently happens that a person recovering from a long illness cannot regain strength without a change of air. At almost every considerable seaside town in England there is a convalescent home, where invalids with small means can spend a month at very little expense; but in the colony he must either go to an hotel, which may be beyond his means, or he must take a house, which includes taking servants, and having all the paraphernalia and worry of housekeeping. Apparently, no convalescent home exists in the colony; neither does a home for inebriates, or a resting-place for governesses out of employment, or an institute for the blind, or for the deaf and dumb.

Again, it is undesirable, for obvious reasons, that idiots, especially idiot children, should be placed in lunatic asylums; yet it is sometimes very hard to keep them at home, and impossible to give them any training. A beginning has been made in this direction at Grahamstown, where a cottage hospital has been built within the precincts of the asylum, where about twelve idiot children are cared for and properly trained. This excellent idea needs to be carried out on a much larger scale.

Another thing very much needed is a hospital for incurables. Incurable patients, who may perhaps linger for years, cannot be taken into general hospitals, and it often happens that very great difficulty is experienced in nursing them at home. Private nurses do not like such cases, because, for one thing, they do not gain the experience they want, and for another thing, the strain, and the monotony of these sometimes very trying cases, is too much in the long run for any one person to bear. Moreover, private nurses, when obtainable, are somewhat expensive luxuries.

There is, both in Capetown and in Grahamstown, an institution called the Chronic Sick Hospital. In Grahamstown it is in connection with the asylum, and although the two buildings are about three miles apart, they are in charge of the same doctor, the Chronic Sick Hospital having also a resident superintendent. In this institution are received old people, both white and coloured, who are helpless, crippled, or paralytic, or suffering from senile dementia. Other cases are also admitted, as, for instance, a few harmless lunatics for whom there is no room in the asylum, or some who have been discharged from the asylum and are not quite fit to go home, or have no homes to go to, and so forth.

It may be thought that these institutions answer the purpose of a hospital for incurables, but they do not.

There are many cases for which it is inadequate. It is practically a pauper institution, and not suitable for those who have always been brought up in comfort and refinement. And there is another point in regard to this and similar institutions. Where all the patients are old and helpless, and unable to work, it follows that all the work of every description falls upon the nurses. All the scrubbing, sweeping, and dusting of the large wards, the making of innumerable beds, washing of utensils, &c., must be done by the nurses, in addition to the charge of their patients, many of whom require to be washed, dressed, and fed like children. In short, a nurse is little but a housemaid, and by the time all her menial labour is done she can have but little time or strength to spare to her patients, and none at all to sit down and talk to them, or amuse them, or study their idiosyncrasies for her own and their benefit.

This difficulty might be removed by having ward servants to do the actual scrubbing, sweeping, and so forth, thus setting the nurse at liberty to devote herself entirely to her patients. A better class of nurses might also be thus obtained. In every institution of the kind the matron, or head nurse, should be a lady, a woman of education and refinement, who could discover and appreciate the same qualities in any of the patients under her charge, and would be able, in many ways, to lighten their lot.

With regard to the lack of institutions in the Colony, what is wanted is private enterprise. I have never heard the Cape Government accused of illiberality in this respect, and what the Government has already done needs to be supplemented by individual effort. We hear of millions that are made in the colony, but we do not hear that any great portion of them is spent there. At all events, it is certain that any wealthy person who seeks a new field for his charity, will have no difficulty in finding one here.

### Dress and Uniforms.

By a Matron and Superintendent of Nurses.

### THE MELBOURNE HOSPITAL.

This important hospital is fortunate in having recently secured the services as lady superintendent of Miss Farquharson, who for five years previously had done such excellent work at the Alfred Hospital. As will be seen by our illustration, the nurses here exhibit the same high characteristics of training with which we were so much struck in the sketch of that hospital. The reason is apparent, however, when we explain that many of the best nurses now at the Melbourne Hospital, having been trained by Miss Farquharson, followed her there when she became its matron. The sisters at the Melbourne Hospital wear a uniform of plain grass-cloth made quite simply, the skirt full, and just touching the ground. The bodice, which fits neatly to the figure, buttons in front, and is united with the skirt by a band at the waist. Over the dress a white linen apron is worn, with a high bib ending in straps which cross and fasten at the back. Deep linen cuffs and collars relieve the bodice becomingly at the wrists and neck, and the plain coronet-shaped muslin cap fits the head neatly and makes an attractive finish to a very pretty costume. The pupil nurses wear a haircard print of a charming lilac shade. It is made quite plain and fits tightly to the figure. A large white linen apron covers the dress furnished with a bib and straps that fasten at the waist behind. The mob cap which completes this dainty uniform is made of plain white muslin with a gophered frill edging.

SISTER. PUPIL NURSE

### Nurses in 1896—Their Quarters, Hours, and Food.

### GUY'S HOSPITAL.

### I.—Terms of Training.

The system of training followed at Guy's Hospital seems hardly in accordance with the modern conditions of nursing. The rules were framed in the days when, in order to secure the right kind of woman to fill the important position of ward sister it was necessary to attract ladies who could afford to pay, and who were of a widely different class from the rank and file of the nursing staff, and still a hard and fast line is drawn between the probationary nurses, who sign for a term of three years, are paid, and are eligible only for appointment as staff nurse, and the lady pupils, who are bound for one year's training only, at the expiration of which they receive a certificate, and are eligible for appointment as ward sister. The duties of the lady pupils in the wards are different from those of the probationer nurses. They are practically trained to take sister's work without going through the whole routine of nursing, accompanying the sister on her round with the doctors, and being accorded various privileges, unknown to the ordinary probationer, amongst others, off-duty time every day. According to the printed rules, it will thus be seen that at Guy's, while it is considered desirable for the staff nurses to receive three years' training, the women who may supervise the whole work of the wards as sisters, and under whose direction these nurses are placed, may themselves have received but one year's training. As the regulations now stand at Guy's only women who can afford to pay can obtain the position of ward sister. Lady pupils are not expected to take night duty during their year's training. Applicants are eligible as nurse probationers between the ages of 23 and 32, as lady pupils from 24 to 32. Nurse probationers enter the hospital on a month's trial. Both lady pupils and nurse probationers are required to attend the regular courses of lectures held by members of the medical and surgical staff, and to pass written examinations thereon. The elements of pharmacy and dispensing are also taught, and examinations held on this subject.

### II.—Hours of Work and Times Off Duty.

Ordinary probationers and staff nurses go on duty in the wards at 8 o'clock, leaving them at night at 9.30. They are only off duty every other day for two and a-half hours; thus, subtracting three half-hours for meals, they are on duty in the wards every other day for twelve hours. They are called at 6.30 a.m., and are expected to be in bed by 10.45 p.m., thus having seven and three-quarter hours in bed. Night nurses go on duty at 9.30 p.m. and go off at 8.45 a.m. They are called at 8 p.m., and are required to be in their cubicles by 1 p.m. There are many distinctions made between the probationers and nurses and the lady pupils; the latter are off duty every day, alternately for one and a-half and two and a-half hours. They are also not expected to return to their ward after supper for half an hour, as the probationers and nurses have to do, and their work is somewhat different in character, as already mentioned. Probationers and nurses are off duty every other day from 2 to 4 30; those who cannot be spared at this time have leave from 6 to 8.30 p.m., having to return to supper and be in their ward again at 9. During their first three years probationers on day duty are only allowed a half-day once a month, afterwards a whole day monthly. On night duty, once a month they are off duty from 10 a.m. one day till 1 p.m. the following day, but are not permitted to sleep away from the hospital except with special permission, being required to report themselves at 9.30 p.m. Probationers have a fortnight's holiday at the end of their first year, three weeks during the second year, and one month afterwards. Sisters and lady pupils are once a month off duty from 4 o'clock on Saturday afternoon to 10 o'clock on Monday morning. At the end of six months' training lady pupils are allowed three weeks' holiday. Sisters have one month's leave in the summer and a week in the winter.

### III.—Meals.

Breakfast for the day nurses is at 7.30, consisting of tea, coffee, or cocoa, bacon or fish, sausages, eggs, bread, and butter. Dinner is at 11.30 and 12, for which half an hour is allowed. For dinner there is meat, with two vegetables, puddings and tarts, and milk or beer. Tea, laid in the dining-room, is from 4 to 5 o'clock. Those nurses or probationers who are off duty at that time are allowed to make their own tea in their sitting-rooms, where dainty tea-services are kept in a special cupboard, and a gas kettle for this purpose is just outside their door. The tea and bread and butter they fetch from the dining-room. This is a pleasant little relaxation, much enjoyed by the nurses. Supper at 8.30 consists of cold meat or soup, cheese, bread and butter, milk, or beer. Night nurses breakfast at 9.10 p.m. (for which twenty minutes seems rather a short time), and take with them to the wards materials for their night meals, usually eggs, and other easily prepared food. Night nurses dine at 9.30 a.m., dietary the same as for day nurses, and there is light luncheon at 12 for those who wish it before going to bed. The tables in the dining rooms are neatly laid and made bright with pots and flowers. Miss Nott-Bower has effected many reforms in this department, and done much to improve the standard of comfort for her nurses.

### IV.—Salaries and Uniform.

Probationers are paid at the following rates: £8 the first year, £12 the second, £18 the third, rising when appointed on the staff to £30. Uniform is provided by the hospital. Two stuff dresses for outdoor wear, with summer and winter cloaks and bonnets, also three print dresses in the year. The sisters are provided with two outdoor dresses and three dark blue linen dresses in the year, with cloaks and bonnets. Lady pupils pay thirteen guineas a quarter for a year's training, and are expected to provide their own indoor and outdoor uniform and pay for their own washing. The washing for the rest of the staff is done in the hospital laundry. It is a pity that the lady nurses do not have washing dresses, their indoor uniform being of black alpaca.

### V.—Nurses' Quarters.

What is called the Nurses' Home at Guy's Hospital only accommodates the lady pupils, but the attainment of a home for all the staff is soon to be no longer a vision of the future but an accomplished fact. The quarters allotted to the nurses and ordinary probationers on the top floor of the medical building are now out of date. They are cubicles closed in with curtains, and at night they are lighted by gas jets down the centre. The furniture, too, is old and the bathroom accommodation scanty; in fact, the provision of proper quarters for the whole of the nursing staff is one of the first reforms needed at this great hospital. We are glad to hear that a new Nurses' Home is about to be erected. The sisters' rooms off their wards, and the lady probationers' rooms have lately been greatly improved, redecorated, and modernised, under Miss Nott-Bower's capable superintendence. The nurses' and sisters' dining-rooms are on the ground and basement floors of the medical building. The former is a fine room, and both have been greatly improved during the last year or two, and are as pleasant and comfortable as can be under existing circumstances. It is a great disadvantage that the nurses' quarters are so scattered. The nurses' and probationers' sitting-rooms, on the right of the main entrance, are cosy rooms, though small for the staff of so large a hospital.

### A Book and its Story.

### "SIREN VOICES."*

If the plot of this story is a slight one, that by no means detracts from its praise. Jacobsen is a remarkable writer, an original thinker, and his works teem with suggestiveness It is not alone what he says, but what his sayings suggest, that places him high among modern novelists. He was not a prolific, but he was a very conscientious writer, a man of deep thought and reflection, and his manner of conveying these thoughts is for the most part a convincing one; even when we are not in sympathy with his views we feel the sincerity of the mind which advances them. "Siren Voices," as has already been said, does not depend for its value on the plot, but on the manner of its telling. It is the story of a young Dane (Jacobsen confines himself to his own country), and the young Dane was to be a poet. "The outward conditions of Niels Lyhne's life had, in themselves, been such as to lead his inclinations in this direction, and concentrate his faculties on the task. Until now, however, he had had little more than his dreams as a basis for his poetry, and nothing is more uniform or monotonous than imagination, for in the ever-changing land of dreams that seems to us so indefinite, there are, in reality, certain short, beaten tracks along which all journey, and beyond which they never stray. People may be very different from one another, but their dreams are always similar, for, in them, without fail, they possess the three or four things they desire, and, no matter whether these things become theirs at once or only gradually, completely or imperfectly, they always get them all; no one really goes empty-handed in his own imagination."

The young man's romantic nature led him into a succession of disjointed love affairs, which form the "motif" of the story. Had he been English, his friends would have dubbed Niels Lyhne with a familiar epithet—he would have been called, perhaps, a flirt, but the nationality of Jacobsen's hero defends him here, as the word flirting is confined to the English and such as use their language.

Niels was in reality a very serious-minded young man, who flung himself heart and soul into whatsoever his right hand found to do, and thus, in his relations with the feminine world, he is equally serious and intense. His friendships are almost all of as absorbing a nature to the reader of the novel as to the hero himself. One of these, for instance, wherein the fascinating Fru Boye plays the leading rôle, particularly compels our interest.

Up to the time Niels meets the lovely little widow his poetry had been in general the fruit of an æsthetic personality, "which," we are told, "found spring a time of buds, the sea great, love erotic, and death melancholy." He had advanced no further in the art of poetry than to be able to string verses together. But now things assumed a different aspect. "Now that he courted a woman's favour and wished her to love him."

Suddenly, in the impassioned earnestness of the young poet's aspirations, he is summoned to his home upon the death of his father, and here, if nowhere else, Niels plays the part of a hero—not through the fulfillment of his dreams of love or verse, but through what may have seemed to him a lower, but, in reality was a higher thing, the bringing of a gentle blessing to the widow, on whom her bereavement told in a fatal manner. Her son's devotion is very touching, very beautiful, from her came the son's inheritance of the poetic, artistic nature. In Fru Lyhne's case it took the form this time, of a craving for the beautiful which amounted to morbidness. "I do not want to die," she sighed to herself. "Do you know what I thought about in those long sleepless nights when death was so frightfully near? What seemed to me harder than anything was that there were so many great and beautiful things in the world I should die without having seen."

Niels tried to comfort her. He made bold plans how, in a short time, as soon as she was well, they should go away together and find the spring in sunnier climes.

And despite all difficulties, the son's zeal carried through his determination, and they journey south; but we are told that her craving after the fairer scenes neither impressed her as powerfully, nor absorbed her as intently as she had imagined it would. She had expected it to be quite different. . . . "And when spring came on its triumphal procession through the valley, with its miracles of germination and its gospel of budding leaves, it had to leave her standing alone, withering in the midst of all this lavish transformation. The new face that streamed from light and air, from earth and moisture, brought no strength to her; her blood felt no healthy craving to join in the universal rejoicings over the omnipotence of spring." . . . And so she died, and Niels buried her in the pleasant cemetery at Clarens, where the brown vineyard soil covers the children of so many lands, "and where the broken columns and veiled nuns repeat the same words of grief in as many different tongues."

The son's devotion to his mother is pathetically, masterfully described; he had renounced his own personal ambition in ministering to the dying woman's wants, and laid aside a companionship which had become unutterably dear to him, that of Fru Boye. And now, on his return to Copenhagen, his first thought is of this lost companionship, and the youth is filled with a wild yearning to see her again, to feel the nearness of her presence.

She sees him, and tells him she is engaged to be married, and that her life will be a different one now—more conventional, less Bohemian. Of her future husband she said, "He is much respected, *very* highly respected; they all wished it so much, and now, you see, I can take up my old position in society again, indeed, if anything, a better one, for he is thought so much of in every respect, and that has been my wish for a long time now." Three weeks later Fru Boye was married, and Niels was left entirely to himself, without the companionship of the woman he had devoutly respected. In whatever way she had chosen to desert him, one thing was certain, he was now alone, and he felt the loss bitterly.

Later on, however, it was a relief to him. So much was waiting for him; "however deeply it had engrossed him at the time, the year spent between Lönborggaard and foreign lands had been an involuntary pause, and the clearer insight into his merits and deficiencies that he had gained during this year had only increased his desire to make use of his talents in the undisturbed seclusion of work." Niels threw himself with enthusiasm into his new labours. He was seized by that desire for conquest, that thirst for the power of knowledge, which every servant of the mind, "however humble his subsequent work, has felt at least once, were it only for a single short-lived hour." And here we will leave the hero of Jacobsen's "Siren Voices," whilst he is still also our hero, too. The book is one of quite unusual masterfulness and interest, but it leaves behind it—more is the pity—an unsatisfactory effect upon the mind of the reader of the futility of all human effort, in whatsoever sphere it may be employed, of ideals, only raised to be shattered, and strength only given to decay. In this tale of Jacobsen's book the man of purpose speaks, the philosopher, the artist, and the poet, but dominating the whole is a sense of hopeless pessimism sufficient to detract from a work of lesser merit than "Siren Voices."

* "Siren Voices." (J. B. Jacobsen. London: William Heinemann, 1896.)

### A Nurse's Visit to Texas.

I.

A few years ago I spent seven months on a small up-country ranch in Texas. Perhaps it may interest some of my fellow nurses to hear a little of what life is like out there for English settlers, especially for women. Their side of the question has not been written of so often as the men's, and as just now I am debarred by ill-health from active work I do not think I could spend my leisure better.

My only brother had gone out on account of lung trouble, and settled there with his wife two and a-half years before, and it had been a promise on my side that I would visit him some day; so when my own health failed in the same way, he renewed his invitation, and I accepted. I found two other ladies who wanted to visit brothers in Texas, so we made up a party, and sailed from Liverpool in the month of September by the s.s. "Britannic," White Star Line; our voyage was much like all others, but it was my first, and I enjoyed it immensely in spite of two days' imprisonment in my cabin through sea-sickness and pouring rain, for we came in for the equinoctial gales, and had a grand tossing.

I was taking charge of a working lad travelling in the steerage, so when I reached New York I went off to Castle Gardens, where the emigrants are landed, to look after him, This lad had been brought up by the Waif and Stray Society since he was seven years old, and he wanted to go abroad, so I had engaged him to help on the ranch for a certain time, in return for his passage out. I may as well mention here, that after fulfilling his engagement he found work on another ranch, and I heard a year ago that he was doing very well.

While I was at Castle Garden Buildings I talked to some people who were expecting emigrant friends and relations, and was very much interested in hearing what good arrangements have been made to protect friendless young emigrants landing in the States. No young woman alone, and no lad under twenty-one is allowed to leave the building until claimed by relations or friends. If not claimed they are kept at the Emigrant Society's Home until places are found for them.

The authorities are also very particular what kind of emigrant they allow to land and settle in the States, for while I was waiting in one of the offices, a man who looked as if he were in very good circumstances was brought in with his two sons. It appeared that the father had been in the States for some time, and had lately sent for these two lads to join him. The younger one was so foolish-looking that he had attracted the attention of the officials, and they objected to pass him. The father asserted that he was quite as bright as most lads. The boy himself did not seem in the least to take in that his wits were in question, for he looked quite unconcerned, and when I left the office it was still undecided whether his father should be allowed to keep him, or would have to send him back.

We took some tram and elevated railway trips to see a little of New York. Visitors to New York should be warned against taking unnecessary cabs. At some of the hotels they will find you one and enter the charge in the bill. Then you are all right, but once when I could not get one that way, and was obliged to hire on my own account, I was charged 12s. for a distance that only took 20 minutes. We had been advised to travel by water from New York to Galveston, the chief seaport of Texas, on the Gulf of Mexico, so we sailed next day in the s.s. "Alamo"—Mallory Line—a pretty little boat, on which we had deck cabins, and were very comfortable, having beautiful weather, tropical sunshine, and the Atlantic as smooth as a lake.

After a week's voyage we landed in Galveston, spent there one night, and the next morning started by train for our several destinations. Mine was Austin, chief town of Texas, with its splendid Capitol, where the State Senate meets.

My brother met me at Austin, and from there to his ranch—a distance of thirty-five miles—we drove in his farm waggon over a road which was so rough and hilly, that I can only compare it to the ascent of Snowdon, on the Llanberis side. We did not get home until after dark, and I was tired enough after my twenty days' journey, and my long jolting drive, to be very glad to have my supper, and slip into the little camp-bed in the sitting-room, where I was to sleep during my stay.

### Novelties for Nurses.

### COOKERY TIME TABLES.

In compiling this useful accessory to the kitchen, Mrs. C. Brooke has really conferred a boon on the community. It is quite astonishing the amount of ignorance that prevails amongst cooks and housekeepers as to any definite measurement of time required in cooking. Experience, which is a very unreliable teacher to many, is in most cases the only guide. Hence the distressing destruction of good food so very common in the poorer households in England. Ignorance of such simple initial details of cooking is responsible for a large amount of discomfort in family life, and in case of sickness is a positive danger. Mrs. C. Brooke's tables should be of material assistance in the kitchen. They are printed in large type on cards, framed in a simple cloth-covered frame, made to stand or hang. In our opinion, for the wear and tear of constant use, a wooden frame should be substituted. The cards are six in number, and are arranged in the order in which the articles of food are served. One card apiece is devoted to fish, meat, poultry, and vegetabless and two to puddings. They are intended to show at a glance the average quantity of each article of food mentioned required for three persons, and the length of time necessary for cooking. The time per pound for larger amounts is also given. Of course, in all matters dependent on varying heat and the conditions of the food, it is impossible to dictate with absolute exactness, but an approximate guide is far better than none, and will enable individual experience to be far more intelligently utilised. Where gas is employed for cooking, the difficulty of calculating is infinitely minimised, as the heat is capable of the nicest regulation, and cooking can be reduced to much a more exact science than could be provided for in tables arranged to meet the average conditions of the majority of households In cottage hospitals and small institutions where the employment of skilled cooks is not always possible, the tables should be welcomed, as they are admirable for reference, and we think they should find a place in every kitchen.

### Minor Appointments.

Whitchurch Cottage Hospital.—Nurse Edith Williams, of Aintree, Liverpool, who holds a certificate from the Rotunda Hospital, Dublin, has been appointed maternity nurse at this hospital. Nurse Mildred Monk, who has received her training at this hospital and at the Sheffield General Infirmary, succeeds to the post of district nurse.

Hull Sanatorium.—We regret that in mentioning last week the appointments of Miss Williams and Miss Whillock as ward sisters at this institution, the latter name was misspelt Whillcock. We would impress upon our correspondents the necessity for writing names very clearly in such communications.

### For Reading to the Sick.

### THE GOOD SHEPHERD.

**Motto.**

"I am the Good Shepherd."

**Verses.**

I was a wandering sheep,
I did not love the fold,
I did not love my Shepherd's voice,
I would not be controlled.
I was a wayward child,
I did not love my home,
I did not love my Father's voice,
I loved afar to roam.
The Shepherd sought His sheep,
The Father sought His child.
They follow'd me o'er vale and hill,
O'er desert, waste, and wild;
They found me nigh to death,
Famished and faint and lone,
They bound me with the bands of love,
They saved the wandering one.
Jesus my Shepherd is,
'Twas He that loved my soul,
'Twas He that wash'd me in His Blood,
'Twas He that made me whole;
'Twas He that sought the lost,
That found the wandering sheep;
'Twas he that brought me to the fold,
'Twas He that still doth keep.

—*Hymns Ancient and Modern.*

Father cf all, to Thee
We breathe unuttered fears
Deep-hidden in our souls,
That have no voice but tears;
Take Thou our hand, and through the wild,
Lead gently on each trusting child.—*Julian.*

**Reading.**

"The Good Shepherd;" well can the sheep who know His voice attest the truthfulness and faithfulness of this endearing name and word. Where would they have been through eternity had He not left His throne of light and glory, to travel down to this dark valley and give His life a ransom for many? Think of His love to each separate member of the flock—wandering over pathless wilds with unwearied patience and unquenchable ardour, ceasing not the pursuit "until He finds it." Think of His love *now*—"I am the Good Shepherd." Still that tender eye of watchfulness following the guilty wanderers—the glories of heaven and the songs of angels unable to dim or alter His affection—the music of the words at this moment coming as sweetly from His lips as when first He uttered them—"I know my sheep." Every individual believer—the weakest, the weariest, the faintest—claims His attention. His loving eye follows me day by day out to the wilderness, marks out my pasture, studies my wants, trials and sorrows, and perplexities—every steep ascent, every brook, every winding path, every thorny thicket, "He goeth before them." . . . "The Lord is my shepherd; I shall not want." I shall not want! It has been beautifully called "the bleating of Messiah's sheep." Take it as thy watchword during thy wilderness wanderings till grace be perfected in glory.—From "The Mind and Words of Jesus."

"Fear not, little flock; it is your Father's good pleasure to give you the Kingdom."—*Luke* xii. 32.

He points His crook upwards to the bright and shining gates of glory, and says, "It is your Father's good pleasure to give you these." What gentle words! Gracious Saviour, Thy gentleness hath made me great!

### Appointments.

### MATRONS.

Stratford-upon-Avon Joint Hospital.—Mrs. Ellis has been selected to fill the post of Matron at this hospital. Mrs. Ellis's previous experience has been at the Fountain Fever Hospital, Lower Tooting.

### Notes and Queries.

**The contents of the Editor's Letter-box have now reached such unwieldy proportions that it has become necessary to establish a hard and fast rule regarding Answers to Correspondents. In future, all questions requiring replies will continue to be answered in this column without any fee. If an answer is required by letter, a fee of half-a-crown must be enclosed with the note containing the enquiry. We are always pleased to help our numerous correspondents to the fullest extent, and we can trust them to sympathise in the overwhelming amount of writing which makes the new rules a necessity. Every communication must be accompanied by the writer's name and address, otherwise it will receive no attention.**

**Queries.**

(180) *Maternity Training.*—Can you tell me of any hospital or infirmary in London or suburbs where free training in maternity work can be obtained?—*Nurse Maud.*

(181) *Sanitary Inspectorship.*—I wish to train as a sanitary inspector. How should I qualify for the work?—*Cornet.*

(182) *Training.*—Please tell me how I can get into a hospital as a probationer. I am in service now, would that be an obstacle? Would it be well to begin as a ward-maid? I have had a little hospital experience in Tasmania.—*Probationer F. G.*

(183) *Monthly Nurse.*—Please advise me. I was engage by a lady for the last week in September, and arranged to call before the time. I write September 1st to say I will call that week, and in reply receive a letter from the lady saying that in consequence of having failed to call she has engaged another nurse. I have refused three cases from doctors for that month. What steps can I take to obtain any recompense?—*M. A. J.*

(184) *Quotation Wanted.*—What quotation would suggest itself to you as descriptive of the enclosed cuttings taken from the correspondence columns of a weekly journal?—*Controversial.*

(185) *Lifting.*—(1) Please tell me if you think it should be the duty of a permanent private nurse to help a butler to carry a male patient up and down stairs? (2) Also is the training at an infirmary such as Lambeth or Lewisham as good as at a general hospital?—*A. F. G. C.*

(186) *Advice Wanted.*—I am anxious to obtain a post as sister of a hospital ward or matron of a cottage hospital, but find a difficulty because I do not possess a certificate from a general hospital. I have had eight years' experience in nursing, four as probationer and then nurse at a provincial women's hospital, then for three years worked in connection with a private and district nursing association. and for the past year have been head nurse of an infirmary block of 130 beds, having good testimonials in each case, and a midwifery diploma. I am 33. Please tell me (1) if I am too old to be admitted for training at a London hospital? (2) Would my eight years' work be a bar to my admission? and (3) Would you consider such a step necessary or worth while?—*Edith.*

**Answers.**

(180) *Maternity Training* (*Nurse Maud*).—See Answer 175 in last week's Mirror. You cannot get free training in midwifery and monthly nursing.

(181) *Sanitary Inspectorship* (*Cornet*).—See reply to "Enquirer" in this column on August 29th.

(182) *Training* (*Probationer F. G.*).—Read "How to Become a Nurse" (Scientific Press, 428, Strand), and write to the matrons of the various hospitals you will find there mentioned for forms of application and particulars. Many matrons are glad to take as probationers women who have been in domestic service. In most general hospitals you would have to sign for three years' service, and would receive a small salary and uniform. All these particulars you will find in the above-mentioned book. There would be no advantage in going as a wardmaid first.

(183) *Monthly Nurse* (*M. A. J.*).—If the facts are exactly as you state, and you did not lead the lady to suppose you would call upon her before the first week in September, and received no communication from her prior to your letter to her, we think you would be entitled to your fee.

(184) *Quotation Wanted* (*Controversial*).—We think you must mean "It is no use keeping a donkey and braying yourself."

(185) *Lifting* (*A. F. G. C.*).—(1) If the lifting is at all beyond your strength, why not say so to your employers! Surely if, as you say is the case, another arrangement could easily be made, there would not be any difficulty about doing so. If the transport of the patient is necessitated by his illness there is nothing *infra dig.* in a nurse giving her assistance. (2) Excellent training is given at such infirmaries as those you mention. Where the matron and ward sisters are fully-trained nurses the teaching is similar to that given in general hospitals.

(186) *Advice Wanted* (*Edith*).—We gather that your four years' training was only in maternity work, and that you have, therefore, had no general hospital training. If this is so we think it would be worth your while to gain experience in medical and surgical general nursing with a view to future promotion. Many of the best hospitals in London and the provinces take probationers up to 35 years of age, and if you explain your reasons for wishing to begin again at the beginning we do not think your previous work will stand in your way. Our advice would be to take as short a training as will give you the certificate you need—one year or two; it will probably be the latter as few hospitals of standing give certificates for a shorter period. In "How to Become a Nurse" (Scientific Press, 428, Strand) you will find full lists of hospitals to choose from, with particulars of training and the ages up to which candidates are eligible.

## Figures and Tables

**Fig. 37. f1:**
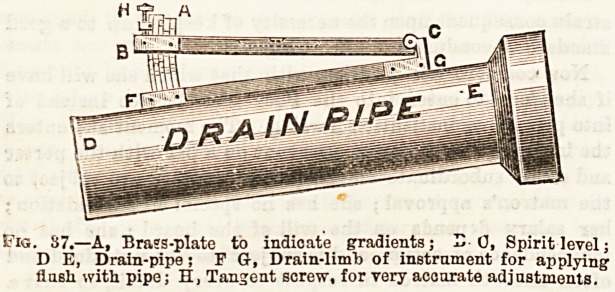


**Fig. 38. f2:**
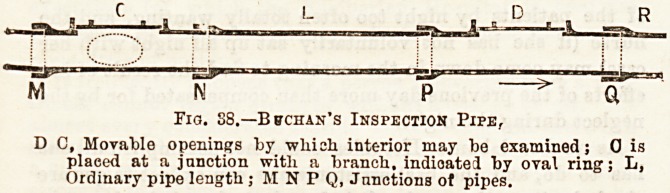


**Fig. 39 f3:**
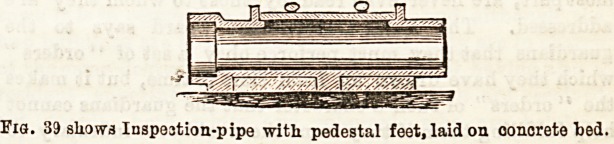


**Fig. 40. f4:**
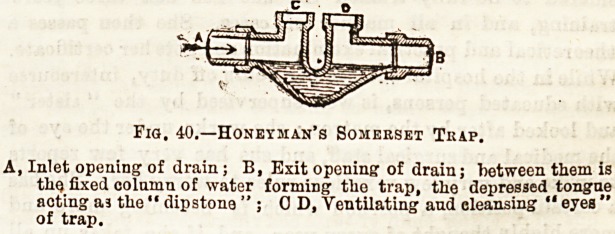


**Figure f5:**